# The Book of Prescriptions

**Published:** 1856-01

**Authors:** 


*The Book of Prescriptions: containing twenty-nine hundred Prescriptions, collected from the practice of the most eminent Physicians and Surgeons, &c.* By Henry Beasley. Phila—Lindsay & Blakison, 1855.

This convenient little formulary, so compact, yet so comprehensive, must prove a handy occupant of the office of the prescriber. It is, indeed, a compendous materia medica, and under each article gives us formulæ, into which enters, in such combination as to secure it most agreeable administration, prompt and pleasant action.

The arrangement is alphabetical, enabling one to turn with facility to any needed medicine, and indeed, in every way, it will be found a convenient, accurate, and reliable formulary. The publishers have gotten it up in a very neat form, not much too large for the pocket even, with good type and paper. The book must succeed.

